# S2O – A software tool for integrating research data from general purpose statistic software into electronic data capture systems

**DOI:** 10.1186/s12911-016-0402-4

**Published:** 2017-01-07

**Authors:** Philipp Bruland, Martin Dugas

**Affiliations:** Institute of Medical Informatics, University of Münster, 48149 Münster, Germany

**Keywords:** Biomedical research, Clinical trials, Database management systems, Data management, Database, Metadata, Model transformation, Statistical data, Software tools

## Abstract

**Background:**

Data capture for clinical registries or pilot studies is often performed in spreadsheet-based applications like Microsoft Excel or IBM SPSS. Usually, data is transferred into statistic software, such as SAS, R or IBM SPSS Statistics, for analyses afterwards. Spreadsheet-based solutions suffer from several drawbacks: It is generally not possible to ensure a sufficient right and role management; it is not traced who has changed data when and why. Therefore, such systems are not able to comply with regulatory requirements for electronic data capture in clinical trials. In contrast, Electronic Data Capture (EDC) software enables a reliable, secure and auditable collection of data. In this regard, most EDC vendors support the CDISC ODM standard to define, communicate and archive clinical trial meta- and patient data. Advantages of EDC systems are support for multi-user and multicenter clinical trials as well as auditable data. Migration from spreadsheet based data collection to EDC systems is labor-intensive and time-consuming at present. Hence, the objectives of this research work are to develop a mapping model and implement a converter between the IBM SPSS and CDISC ODM standard and to evaluate this approach regarding syntactic and semantic correctness.

**Results:**

A mapping model between IBM SPSS and CDISC ODM data structures was developed. SPSS variables and patient values can be mapped and converted into ODM. Statistical and display attributes from SPSS are not corresponding to any ODM elements; study related ODM elements are not available in SPSS. The S2O converting tool was implemented as command-line-tool using the SPSS internal Java plugin. Syntactic and semantic correctness was validated with different ODM tools and reverse transformation from ODM into SPSS format. Clinical data values were also successfully transformed into the ODM structure.

**Conclusion:**

Transformation between the spreadsheet format IBM SPSS and the ODM standard for definition and exchange of trial data is feasible. S2O facilitates migration from Excel- or SPSS-based data collections towards reliable EDC systems. Thereby, advantages of EDC systems like reliable software architecture for secure and traceable data collection and particularly compliance with regulatory requirements are achievable.

**Electronic supplementary material:**

The online version of this article (doi:10.1186/s12911-016-0402-4) contains supplementary material, which is available to authorized users.

## Background

Electronic data collection is a major advance in the conduction of clinical trials compared to paper based documentation [1]. Data capture for observational studies or registries is often performed in spreadsheet-based applications like Microsoft Excel or directly in statistic software like IBM SPSS [2–5]. In any case, data is transferred into statistic software, such as SAS [6], R [7] or IBM SPSS Statistics [8], for analysis. Applications like Excel or SPSS are commonly used in academic research institutions: They are easy-to-use, relatively cheap and provide flexible data structures (variables can be added and removed as needed). In contrast, electronic data capture (EDC) systems are used to collect and manage data for interventional trials in a regulated setting.

In the following, we define data collection tools that are based on spreadsheets like Excel or SPSS as SBDC (spreadsheet-based data collection) software whereas EDC systems are understood as applications for the conduct of clinical trials. EDC systems must comply with regulatory requirements of pharmaceutical regulating authorities like the Food and Drug Administration (FDA) [9] or the European Medicines Agency (EMA) [10]. In contrast to SBDC systems, EDC software is usually used as remote data entry (RDE) system.

SBDC applications can save setup and training time, especially for smaller studies, but this kind of data capture suffers from several drawbacks: Documents are often stored on a local place or network share, not allowing shared access or simultaneous work. Further disadvantages are missing data security in terms of right and role based access control. Backup for SBDC databases is commonly performed manually by copying files to external storages. This may result in version conflicts especially when multiple researchers are involved. Usually, SBDC software does not support the workflow of clinical trials, e.g. event calendars, which are critical for longitudinal study design. Missing traceability of entered data is also a major concern. In this concern, a change log is not available, e.g. it cannot be audited who performed which data changes neither when nor why.

In contrast to SBDC applications, data collection with EDC systems can be managed for multiple users and sites. Central hosting with access via Internet enables trustworthy backups of the latest data including its change history [11]. Access rights and roles can be managed centrally. Due to regulatory requirements EDC systems for interventional trials must undergo a validation process according to regulations for electronic data capture in clinical trials [12] like Good Clinical Practice (GCP) [13] or FDA 21 CRF Part 11 [14]. In contrast to SBDC applications, EDC software is capable to comply with these regulations and designed to support an organized workflow from the creation of forms and the management of queries to the closure of the database.

Nevertheless, the interoperability of commercial and open-source EDC applications varies. Almost all systems are capable to export data as spreadsheet file for transfer into statistic software. In addition, many systems can import clinical values for instance from central laboratories. The Operational Data Model (ODM) from the Clinical Data Interchange Standards Consortium (CDISC) is a commonly supported transport format for EDC systems [15]. ODM is a format for defining the electronic case report form (eCRF), communicating and archiving metadata as well as patient data in clinical trials [12, 16]. Of note, it is capable to store a complete audit trail of captured data. Commercial and academic EDC-solutions like x4T-EDC [17] are able to directly create the trials’ database from the imported ODM data structure.

Pre- or pilot-studies are often conducted before large-scale clinical trials. When these pilot studies are successful, data collection needs to be upgraded to meet the requirements of multi-user and multi-center trials, in particular regulatory compliance, scalability and technical security. Clearly, EDC systems are the means of choice for remote data entry by multiple users and institutions. At present, the change towards an EDC system implies a complete new setup of the study database structure, which is a labor-intensive and error-prone manual process.

To our knowledge, no transformation approach or tool exists to support the conversion and exchange of research databases. Therefore, the aim of our software tool S2O is the conversion between SPSS and CDISC ODM format to foster the transfer of SBDC towards EDC systems, including data transformation. The second goal is to evaluate the conversion process regarding syntactic and semantic correctness and its limitations.

## Implementation

Many statistic programs like SAS and R can export data as SPSS file, therefore SPSS was selected as source data format. This research work is divided into a technical implementation and an evaluation of transformation results. Format specifications were reviewed to develop a mapping model. Based on this model the converter software S2O between IBM SPSS and CDISC ODM was programmed in Java as a command-line tool.

### Technical approach

To implement the transformation of IBM SPSS into CDISC ODM files, the specification of the SPSS file structure and ODM v1.3.1 were reviewed. SPSS is a binary format; so libraries and application programming interfaces (API) are used to access the content.

Another approach in database research is the concept of ‘schema matching’, which is understood as the identification of semantic correspondences between two different schemas [18, 19]. In case of for instance two XML schemas, this technique could be applicable. However, the SPSS schema is proprietary and does not contain semantic annotations.

#### SPSS and available interfaces

Different to Excel or Lotus spreadsheets, SPSS files contain a flat table structure for variable definitions and value lists to specify the dataset. Variable and value labels can be defined in one language. SPSS variables are defined by type (for instance string, numeric or date), width (number of characters), decimals, labels, values, missing values, column, align, measure and role. Column and align are only used for display purposes.

Several libraries are available for use with Java: Two “SPSS-Reader” libraries, SpssJava-Plugin and Talend open Studio. The first “SPSS-Reader” library is available as open-source software and was developed by the Open Data Foundation [20], dated 2008. It does not support the conversion into a directly processible format but rather into a specific format of the Data Documentation Initiative which requires further processing steps. The second “SPSS-Reader” library is available as a commercial product and maintained by pmStation [21]. It allows native access to read variables captured data cases. pmStation also offers a library for writing SPSS files in Java. Furthermore, Talend Open Studio processes SPSS files upon a broad variety of input and output formats [22]. This ETL (extract, load and transfer) tool is available as open-source application for multiple operating systems and allows reading and writing SPSS variables and case data. Scenarios, which are developed within Talend, can also be exported as standalone Java applications. Nevertheless, this plugin is only available as 32bit version and does not support 64bit operating systems. Since version 16, IBM SPSS is based on Java and also available for Mac OS X and different Linux distributions. The SpssJava-Plugin is an internal library of IBM SPSS Statistics for the use in Java programs [23]. It is included in the standard SPSS installation since version 21 and allows reading and writing of variables and case data. SPSS commandos can also be transmitted by this Plugin. Nevertheless, it requires an installed and licensed version of IBM SPSS Statistics on the local computer. Hence, it has the advantage that the software vendor directly supports the latest modifications on the SPSS file format which are continuously included in its development. The IBM SPSS internal SpssJava-Plugin was selected for the S2O application due to the limitations of the mentioned alternatives.

#### CDISC ODM

**Fig. 1:**
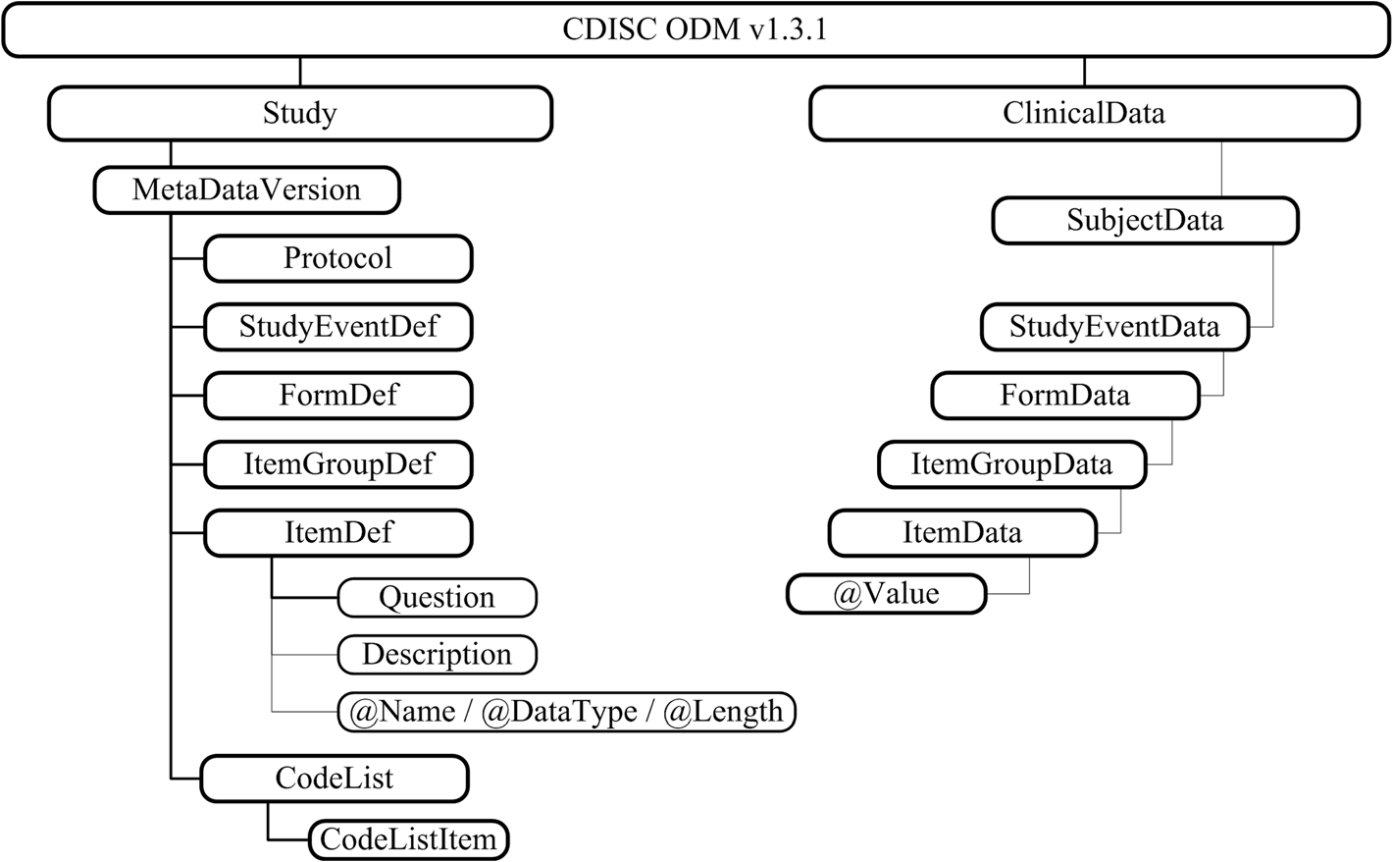
Sections of CDISC ODM with study metadata information left-hand side and structure of clinical values on the right. For metadata there is one hierarchy for elements to reuse them in a study. In contrast, data is hierarchically represented according to the metadata structure

CDISC ODM is an XML-based format that defines the structure of trial eCRFs. *Study-* and *ClinicalData* are the main components of ODM, which consist of study metadata and its associated clinical values. Both elements provide the hierarchy of study events, forms, item groups and items as shown in Fig. 1 (*AdminData*, *ReferenceData* and *Association* elements are omitted to improve readability).

Several versions of metadata can be administrated for a study. All child elements from *Protocol* to *CodeList* appear below the *MetaDataVersion*. A *Protocol* specifies a trial protocol and the *StudyEventDef* defines a set of *FormDef*s usually corresponding to a patient visit. A *FormDef* represents an eCRF and consists of *ItemGroupDef*s consisting of various *ItemDef*s. These items are the specification for a single data element. *ItemDef*s are specified with a name and data type. A *Question* and *Description* can be given as well as a *CodeList* that contains permissible values. Although all child elements are on one layer, the hierarchy is obtained by the use of referencing OIDs. It allows the reuse of *Items*, *ItemGroups* and *Forms* within its parent elements. Furthermore, *ClinicalData* contains the data values for each *ItemDef*. Therefore, the same OIDs are used in the *ClinicalData* and *MetaData*-elements. Data values are stored within the *Value*-attribute of the *ItemData* element. The root element for each patient file is the *SubjectData*-element that contains the *SubjectKey* attribute as patient identifier.

#### Programming

The S2O application is programmed in Java using the Eclipse IDE with Oracle Java version 1.7. The JDOM 2.0.6 library is used to create the converted ODM-XML-structure. S2O is provided as a command-line tool that uses the Apache Command Line Interface version 1.2 to handle parameters with options. The application is exported as JAR-file and must be placed within the IBM SPSS installation folder to access the required library which is included in the standard SPSS installation.

### Evaluation

#### Metadata structure

Nine SPSS files with different complexity were selected to evaluate S20 (see Table 1). Clinical cooperation partners provided these files that contain unpublished data of clinical registries. Provided datasets have been fully anonymized to comply with the data protection regulations and are only indexed by an incrementing number. One sample SPSS file [see Additional file 1] with all available data types and eight files from real clinical studies and registries (S1-S8) were analyzed.

**Table 1:** SPSS input files of different projects and a sample file with all available data types

Project	S1	S2	S3	S4	S5	S6	S7	S8	Sample
# Variables	139	382	455	34	67	24	188	645	16
# Cases	2075	3452	2890	2890	2890	621	0	0	5

Seven of these files contain clinical data; in two only metadata is available. Those SPSS files contain a minimum of 16 variables and five patients and a maximum of 645 variables and 3452 patients. Semantic correctness was validated with the ODMView tool from IPL [24]. This validation inspects the association of ODM elements – for instance the group affiliation of items or item groups in forms – which is covered by OIDs within the element structure. Syntactic correctness was validated by uploading the results into the portal of medical data models [25], which is based on ODM. During the upload process each XML file is checked whether it complies with the ODM schema definition. In addition, the download option as SPSS-file was used to compare the SPSS input file with the result of a conversion to ODM and back to SPSS format.

#### Patient data structure

Converted clinical cases were validated with the in-house developed x4T-EDC system [15]. The metadata of studies S2, S3 and the sample SPSS file was uploaded into x4T-EDC to create the database structure. Then the *ClinicalData* part was uploaded to the respective study. Subsequently, the number of SPSS cases was compared with the amount of imported patients in x4T-EDC. A manual check was performed on the complete sample file and on a random selection of patients from the other two studies.

## Results

### S2O mapping model

**Fig. 2:**
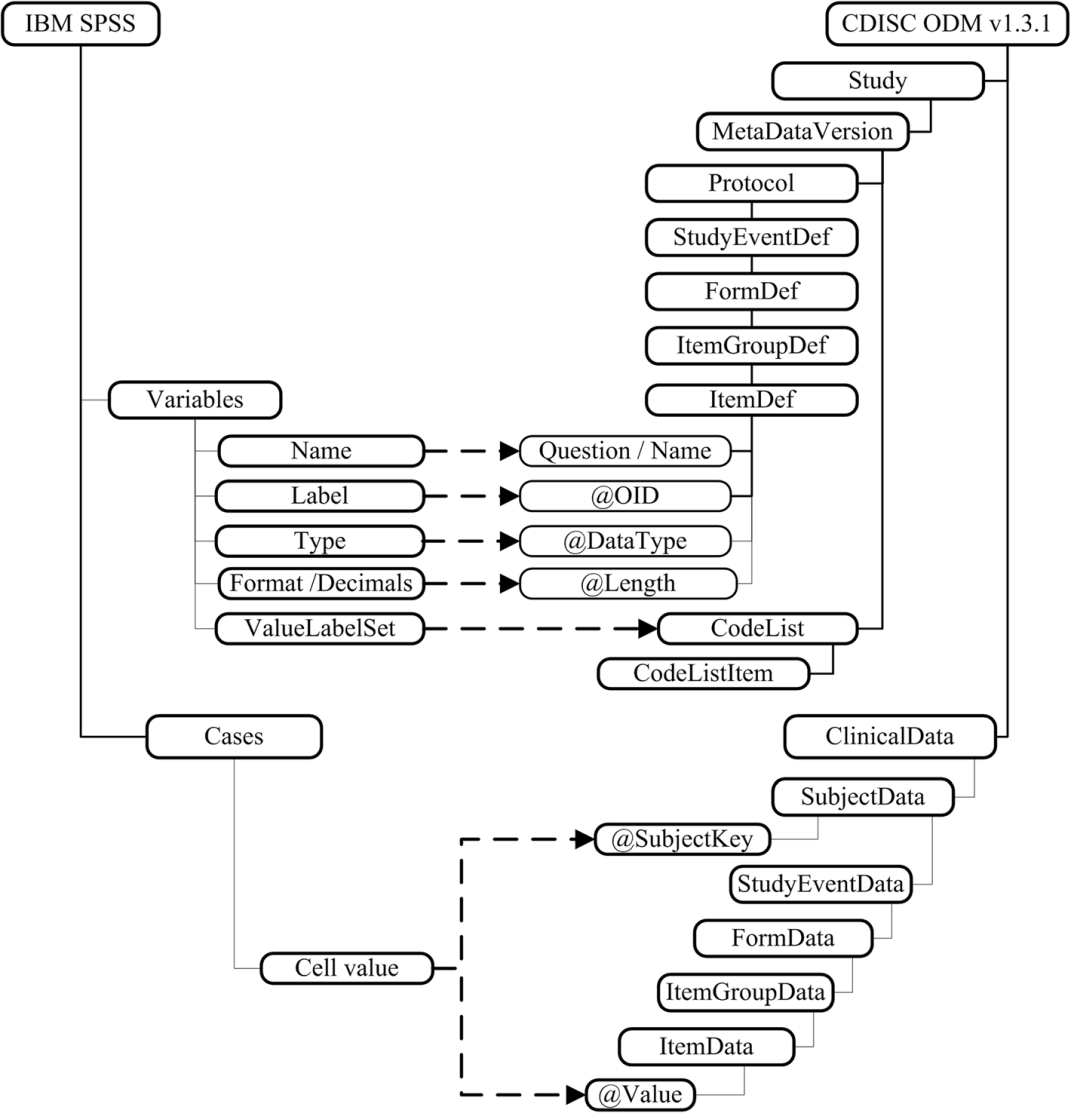
Mapping model between IBM SPSS and CDISC ODM. The upper part describes the mapping between SPSS variables and ODM metadata definitions which are mainly stored within the *ItemDef*- and *CodeList*-element. Clinical cases correspond to the *ClinicalData*-element. Values are stored in the respective *ItemData*-*Value*-attribute which is shown in the lower part

Most elements are transformable between IBM SPSS and CDISC ODM which is shown in the mapping model in Fig. 2. The upper part describes the metadata structure. SPSS does not contain study related attributes concerning protocols or events and variables are defined in a flat list structure. Values in SPSS are entered in one row per case.

Study-specific elements like the protocol, events or forms are not stored in SPSS and are therefore included per default in the ODM structure. The SPSS-variable consists of the attribute Name (maximum length 64 characters), which is corresponding to the *ItemOID* in ODM and Label mapped to the *Question*-element and the *Name*-attribute. For existing SPSS variable-types a mapping to corresponding ODM data types is created. Apart from rarely used date formats like “Week and year“or “Day of the week” all data types can be mapped to corresponding XML-types on a generalized level. Variable width and decimals attributes can also be mapped to the *Length* and *SignificantDigits* attributes of ODM. Permissible Values correspond one-to-one to *CodeList*-elements, including *CodeListItem*-elements. Certain statistical attributes like Missing, Measure and Role are not represented in ODM. The display parameters Columns and Align also cannot be mapped to the XML-structure. SPSS is able to define the date in different formats whereas ODM uses a XML-specific format. Thus, the information regarding the display format will not be included in the resulting ODM file. ODM provides text labels in multiple languages using IETF RFC 3066 language codes [26]. Variable labels in SPSS can only be defined in one language.

In SPSS clinical values are stored in cases, which are converted into the *ClinicalData* element of ODM. In analogy to metadata information, *StudyEventData*, *FormData* and *ItemGroupData* are added by default. Values are stored in the *Value*-attribute of the *ItemData*-element.

### Implementation of S2O

**Fig. 3:**
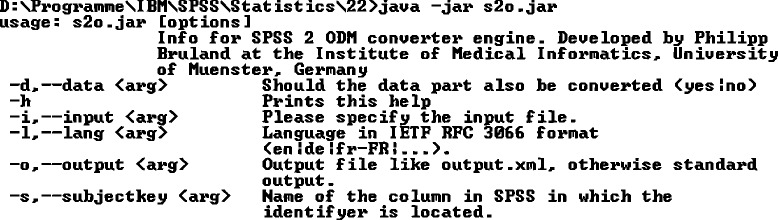
S2O command line application. Input file must be given. All other parameters are optional. It can be chosen whether the data should be converted, which source language is present and which column in SPSS contains the subject identifier

The S2O tool is developed as command-line application shown in Fig. 3.

An input file must be given as parameter that contains the path to the source SPSS file. All other parameters are optional and can be left blank. In this case the conversion result will be directly printed to the command-line. Metadata is always exported, and by the “data”-parameter it can be chosen whether clinical data is also included in the output. The “subjectkey”-parameter points to the patient identifier (case-sensitive) column in SPSS. S2O includes an incrementing number per default as *SubjectKey*-attribute, if this parameter is not specified. Otherwise, this identifier column will not be converted as a separate item. Furthermore, ODM is able to manage multiple languages, which are written into *TranslatedText*-elements for each text that is displayed to the user. To define a language for text in SPSS, an IETF RFC 3066 compliant language code can be stated as “lang”-parameter, otherwise the language information will not be included. Entered language codes are validated to prevent incorrect input.

### Evaluation of S2O

#### Metadata structure

**Fig. 4:**
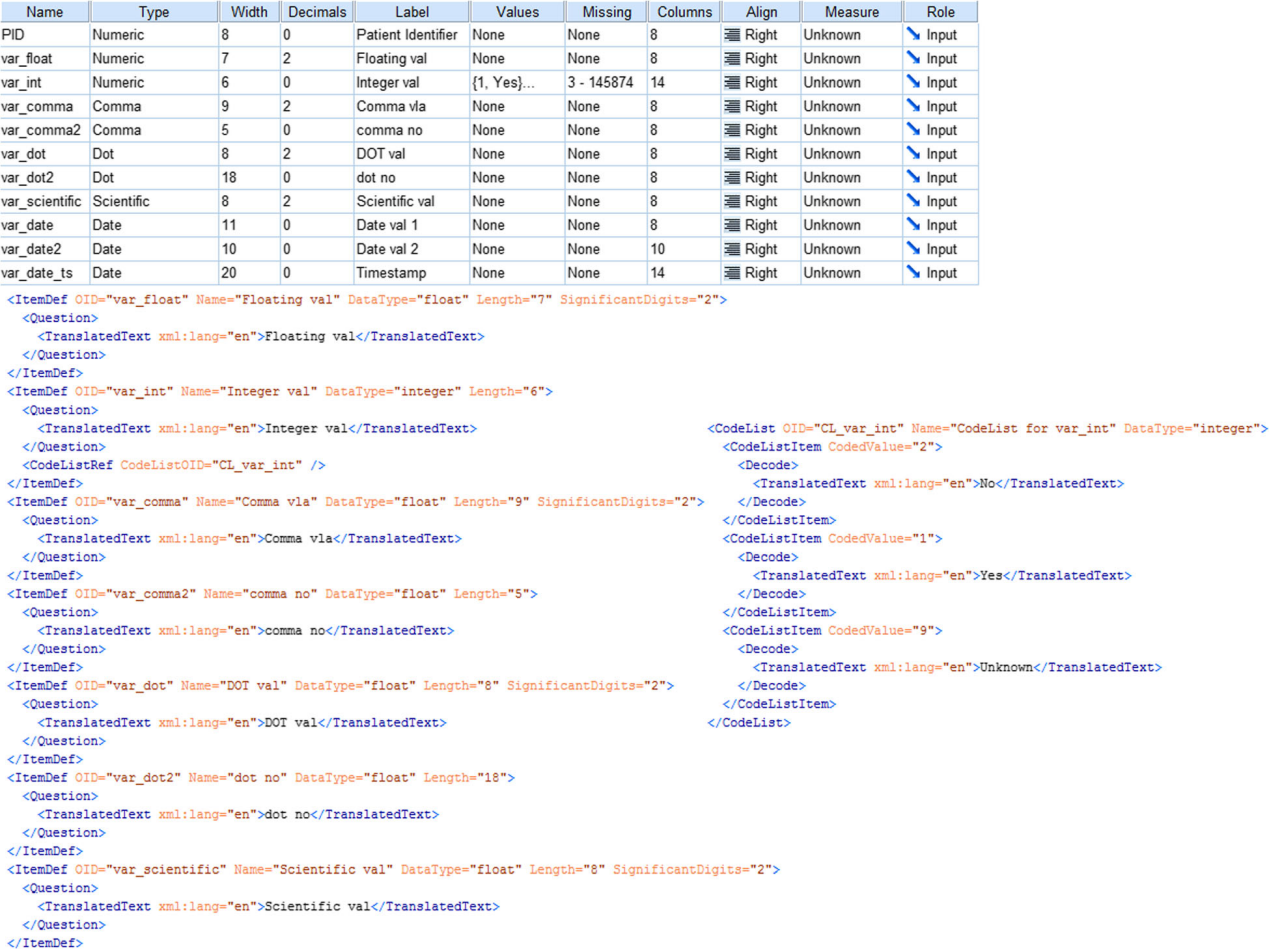
Upper spreadsheet part: Snapshot from SPSS test file is shown in the variable view. Lower XML part: Result of conversion (excerpt) in CDISC ODM. Item definitions and a *CodeList* are presented

To identify the accuracy of the mapping model and the transformation, all study files from Table 1 were converted. The sample SPSS file and an extract of the converted ODM result is shown in Fig. 4. The upper part of Fig. 4 shows the SPSS variables, their labels, data types, value domain and statistical attributes. In the lower part of Fig. 4 the result of the converted ODM is presented.

After the conversion the resulting ODM file was uploaded into ODMView. The validation did not detect any errors, therefore XML syntax and semantics of ODM elements was correct. To discover possible discrepancies in the conversion, the ODM file was again converted to SPSS format: SPSS data types like Scientific, dot, comma, special-integer and currency specific types could only be matched to less specific XML-types. This causes a minor loss of information. In addition, display settings like column and align as well as statistical attributes like measure, role and missing values do not completely match to any corresponding element in ODM. During the conversion from ODM to SPSS they were set to default values. Only the numeric, date, time and string data types can be mapped to ODM, namely string, integer, float, data, time and datetime. Variable labels and values were successfully matched to ODM elements and back to SPSS format.

#### Patient data structure

Syntactic and semantic correctness of the converted clinical values was verified by import into the x4T-EDC system and manual check of values. The ODM metadata part was successfully imported for the S1, S2 and the sample file.

**Fig. 5:**
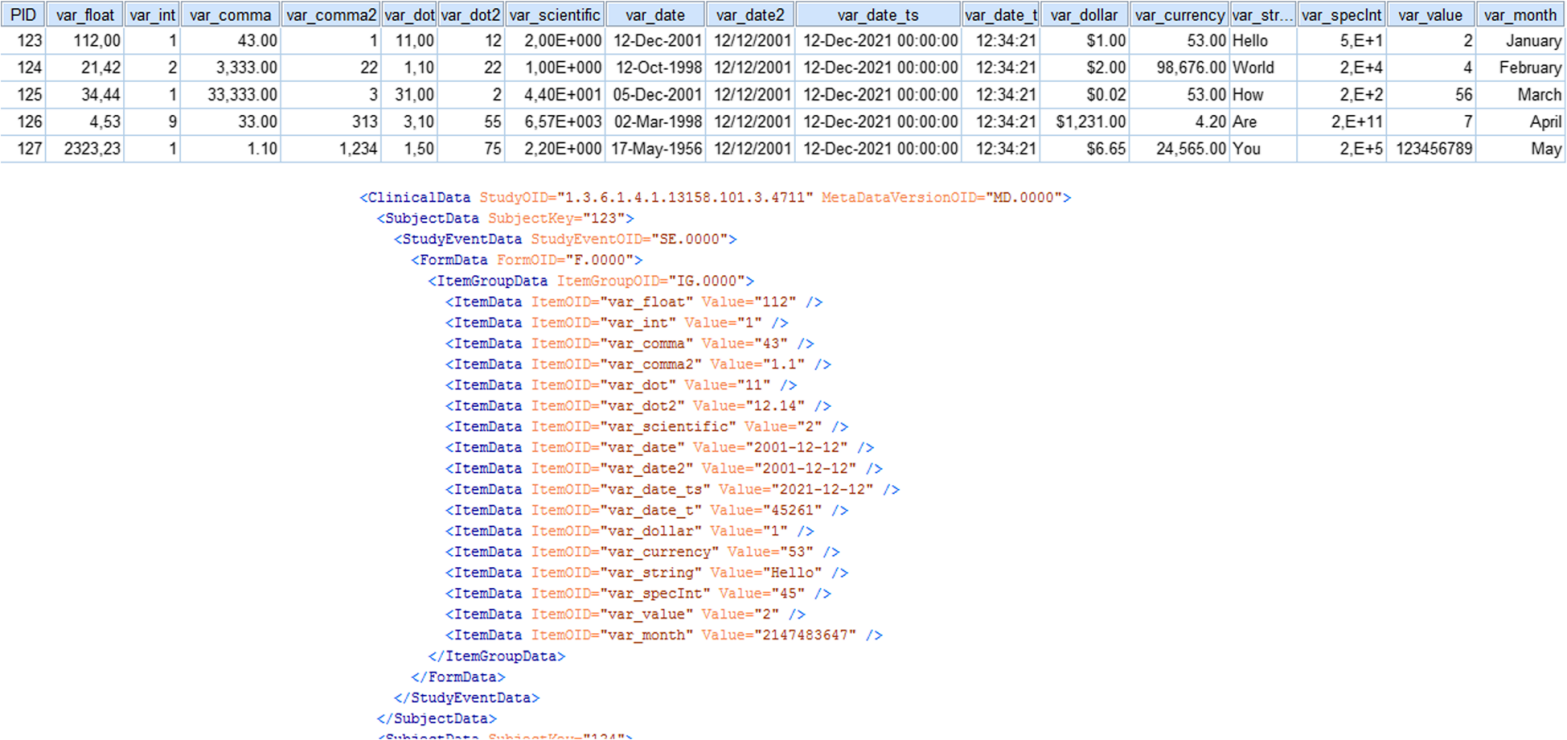
Upper spreadsheet part: List of SPSS cases with respective values. Lower XML part: The resulting ODM *ClinicalData*-part of the first SPSS case

Thereafter, the converted *ClinicalData*-part was uploaded into the system. The upper part of Fig. 5 shows a list of patient test cases. The converted ODM result is shown in the lower part and does not contain the “PID”-column as *ItemData*-element; it is rather transformed into the *SubjectKey*-attribute as patient identifier. All data values were correctly imported and assigned to the corresponding patient identifier variable. The SPSSJava-Plugin transforms SPSS types like DOT and COMMA to usual decimal and integer values which are included in ODM. Although different date formats were specified in SPSS, conversion to ODM results in an XML-specific format for date values [see Additional file 2 for the result of the conversion].

In total, 1991 items from ten studies were processed successfully.

## Discussion

Data transfer between electronic systems for data capture is a crucial functionality. S2O converts the statistical spreadsheet-based format IBM SPSS into a standard format for electronic data capture in clinical trials. The tool supports and promotes the manual transformation process. SPSS is a very popular format and supported by several statistic programs. For instance, statistic courses are held in front of medical students mainly in SPSS to prepare them for performing scientific data collections and different analyses. In addition, SPSS allows importing data from several applications such as Excel or Lotus spreadsheets, STATA, dBASE and SAS. On the other hand applications like SAS or R are capable to export data into SPSS format. For these reasons SPSS was chosen as a source format for the conversion with S2O.

In S2O the IBM SPSS internal library was used for the development of the converter and to access the SPSS values. Promising approaches from database research like schema or ontology matching [18] could not or only tediously be applied since SPSS offers no semantic annotation or ontology capabilities.

When integrating an existing SBDC into a common EDC system, the S2O converter eliminates the drawback of cumbersome and error-prone manual transformation of variables and clinical values by the transformation of SPSS into the CDISC ODM format. Furthermore, it fosters the use of regulatory-compliant EDC systems with key benefits like access for multiple users, data security and traceability of entered data. Nevertheless, data from SBDC applications needs to be examined carefully before upload into EDC systems.

Overall, we would advise researchers to refrain from utilizing spreadsheet software like Excel or OpenOffice and statistics software with spreadsheet-based data collection like SPSS or SAS as a primary tool for data capture in any research project. Open-source EDC systems like OpenClinica [27] or REDCap [28] as well as commercial EDC tools are available and allow importing subject data via ODM. These tools need some efforts but are eligible avoiding problems and drawbacks of SBDC software.

### Strength and weaknesses

S2O covers the transformation of all relevant meta-information regarding SPSS variables and the values itself into the CDISC ODM format. SBDC systems usually contain a flat list of variables, whereas the ODM-format is hierarchically constructed. Hence, data elements of spreadsheets are inserted into a default structure of protocol, study events, forms and item groups in ODM. An automatic recognition of the patient identifier variable in SPSS is not possible. Due to the fact that a subject key must be given in ODM to identify the clinical cases, a parameter in S2O can be used to indicate the SPSS variable name that will not be converted as a separate ODM variable but set as *SubjectKey* to identify the record. Otherwise, if no variable is available or given, a default iterator for subject identification is placed instead.

The mapping of variables, labels, data types and value lists is possible without any detriment. Apart from statistical information, such as role, measure and missing values, the structure of research variables and SPSS data values are fully convertible into the CDISC ODM format.

Depending on the data collection scheme, spreadsheet-based solutions often contain several cases per patient for follow-up visits, which results in multiple rows of data per patient. Currently, the S2O-application is not capable to identify and handle multiple cases per patient. A dynamic list of repeating variables might be applied to include those cases into multiple repeating *FormData* or *ItemGroupData*-elements within the *ClinicalData*-hierarchy. A further minor weakness is the loss of date format and alignment information during the conversion process.

ODM is only able to process the XML-date format and does not store country-specific display formats.

### The role of ODM in electronic data capture

According to the FDA’s Data Standards Catalog, this authority accepts Define-XML as communication format for the definition of clinical study data, which is an extension of the ODM standard [29] and currently, the FDA is performing a pilot evaluation project to identify a new standard for the electronic submission of trial data [30]. This pilot project comprises the evaluation for the applicability of the ODM-Dataset-XML standard (also an extension of the ODM format) as an alternative for the ageing 8bit SAS XPORT format.

ODM on the other hand, is a distinguished standard for exchange and archiving of clinical trial metadata as well as clinical data [10, 31]. With the aid of official CDISC extensions ODM is also capable to process and communicate trial protocol information [16]. Thus, several EDC systems accept CDISC ODM as a data modeling and exchange format, the communication of converted study-related data can be established and fosters the model-driven-architecture approach for creating the trial database. EDC systems usually fulfill the regulatory requirements such as GCP [32]. Metadata from many CRFs in ODM format are available for example in the portal of medical data models

### Clinical data models

Data models in healthcare and research need to be kept interoperable for data exchange between different applications. In this regard, Legaz-García et al. have developed a mapping model between the Clinical Element Model and the openEHR Archetypes [33]. A converter for transformations between CDISC ODM and the Archetype Description Language was described previously [34]. The advantage of this approach is that the data structure is the same in both systems and captured data can easily be merged for statistical analyses. In addition, a mapping scheme for transformations between the ISO11179 standard for metadata registries and ODM was created [35]. This approach has been validated by converting all released CRFs from the NCI caDSR repository and uploading the result into the portal of Medical Data Models. In ODM it is possible to enrich medical concepts with codes of common terminologies. Semantically annotated forms allow comparison and frequency analyses if a large amount of forms is available in a structured way [36, 37]. It has also been shown, that ODM is eligible for the exchange of clinical data between different medical applications for instance electronic health record systems and EDC [38–40] systems or research platforms like i2b2 [41, 42].

### Future work

The aim of a further release of the S2O converter will be the improvement of the algorithm towards the capability to handle multiple rows of values per patient from the SPSS file. Although it is rather a minor limitation, a future release of the converter should work without the SPSS internal library that requires SPSS to be installed on the computer.

An XML vendor extension of ODM could be applied to map the missing SPSS parameters such as alignment, role, missing values or measure. Then it would be possible to establish a full bidirectional conversion.

## Conclusions

Transformation between the spreadsheet format IBM SPSS and CDISC ODM as standard for the definition and exchange of clinical trial data is feasible. The software tool S2O facilitates an accurate conversion between both data standards. SBDC tools like Microsoft Excel or IBM SPSS Statistics do not meet regulatory requirements for data capture. The S2O tool could reduce manual steps for migration of databases to reputable EDC systems.

## Availability and requirements

- Project name: S2O
- Project home page: http://sourceforge.net/projects/s2o
- Operating system(s): Windows, Linux, UNIX Server systems, Mac Os
- Programming language: Java 1.7
- Other requirements: Java 1.7 or higher, IBM SPSS Statistics v21 or higher
- License: LGPL
- Any restrictions to use by non-academics: no, but IBM SPSS Statistics is needed

## Additional files

Additional file 1: Sample SPSS file. The data contains variables with all possible data types and example cases with values. (SAV 3 kb)
Additional file 2: Converted ODM result: The file contains the result of the S2O conversion in CDISC ODM format. It includes all metadata variables as well as clinical cases. (XML 12 kb)

## Abbreviations

API: Application programming interface
CDISC: Clinical Data Interchange Standards Consortium
eCRF: electronic Case Report Form
EDC: Electronic data capture
EMA: European Medicines Agency
ETL: Extraction-transfer-load
FDA: Food and Drug Administration
GCP: Good clinical practice
ODM: Operational data model
RDE: Remote data entry
SBDC: Spreadsheet-based data collection
